# Supplementary material for: Nuclear receptor Ftz-f1 promotes follicle maturation and ovulation partly via bHLH/PAS transcription factor Sim
Source: eLife. 2020 Apr 27;9:e54568. doi: 10.7554/eLife.54568 (PMC7239656; doi:10.7554/eLife.54568)
Supplement: Supplementary file 4. [file elife-54568-supp4.docx]

| **Key Resources Table** | | | | |
| --- | --- | --- | --- | --- |
| **Reagent type (species) or resource** | **Designation** | **Source or reference** | **Identifiers** | **Additional information** |
| genetic reagent (*D. melanogaster*) | *ftz-f1^ex7^* | (Yamada et al., 2000)*.* | FLYB:FBal0120497 |  |
|  |  |  |  |  |
|  |  |  |  |  |
| genetic reagent (*D. melanogaster*) | *ftz-f1::GFP.FLAG* | BDSC | BDSC:38645 ; |  |
|  |  |  | FLYB: FBst0038645 |  |
|  |  |  | RRID:BDSC_38645 |  |
| genetic reagent (*D. melanogaster*) | *ftz-f1^fs(3)^*^2877^ | (Karpen and Spradling, 1992) |  |  |
| genetic reagent (*D. melanogaster*) | *Mmp2::GFP/Cyo* | (Deady et al., 2015) |  |  |
| genetic reagent (*D. melanogaster*) | *Vm26Aa-Gal4* | (Peters et al., 2013) |  |  |
| genetic reagent (*D. melanogaster*) | *Oamb-RFP* | (Knapp et al., 2019) |  |  |
| genetic reagent (*D. melanogaster*) | *47A04-LexA* | BDSC | BDSC:54873 ;RRID:BDSC_54873 |  |
|  |  |  |  |  |
|  |  |  |  |  |
| genetic reagent (*D. melanogaster*) | *lexAop2-6XGFP* | BDSC | BDSC:52265; RRID:BDSC_52265 |  |
|  |  |  |  |  |
| genetic reagent (*D. melanogaster*) | *sim3.7-Gal4* | BDSC | BDSC: 26784 | (Xiao et al., 1996) |
|  |  |  | FLYB: FBst0026784 |  |
|  |  |  | RRID:BDSC_26784 |  |
|  |  |  |  |  |
| genetic reagent (*D. melanogaster*) | *UAS-EcR^DN^* | BDSC | BDSC: 6872 |  |
|  |  |  | FLYB: FBst0006872 |  |
|  |  |  | RRID:BDSC_6872 |  |
| genetic reagent (*D. melanogaster*) | *UAS-ttk^RNAi^* | VDRC | VDRC: 101980 FLBY:FBst0473852 RRID:FlyBase_FBst0473852 |  |
| genetic reagent (*D. melanogaster*) | *UAS-Cyp18a1* | (Rewitz et al., 2010), |  |  |
| genetic reagent (*D. melanogaster*) | *UAS-Cyp18a1^RNAi^* | VDRC | VDRC: 5602 |  |
|  |  |  | FLYB: FBst0470059 |  |
|  |  |  | RRID:FlyBase_FBst0470059 |  |
| genetic reagent (*D. melanogaster*) | *UAS*-*ftz-f1^RNAi1^* | BDSC | BDSC: 33625 |  |
|  |  |  | FLYB: FBst0033625 |  |
|  |  |  | RRID:BDSC_33625 |  |
| genetic reagent (*D. melanogaster*) | *UAS- ftz-f1^RNAi2^* | VDRC | VDRC: 104463 |  |
|  |  |  | FLYB: FBst0476321 |  |
|  |  |  | RRID:FlyBase_FBst0476321 |  |
| genetic reagent (*D. melanogaster*) | *UAS-ftz-f1* | (Yussa et al., 2001) |  |  |
| genetic reagent (*D. melanogaster*) | *UAS-sim^RNAi^* | VDRC | VDRC: 26888 |  |
|  |  |  | FLYB: FBst0456642 |  |
|  |  |  | RRID:FlyBase_FBst0456642 |  |
| genetic reagent (*D. melanogaster*) | *UAS-sim-3xHA* | FlyORF | FlyORF: 000719 |  |
|  |  |  | FLYB: FBst0501520 |  |
|  |  |  | RRID:FlyBase_FBst0501520 |  |
| genetic reagent (*D. melanogaster*) | *UAS-mSF1* | Yussa et al., 2001 |  |  |
| genetic reagent (*D. melanogaster*) | *hsGal4^DBD^-EcR^LBD^, UAS-nlacZ* | (Kozlova and Thummel, 2002) |  | gift by Wu-Min Deng |
| chemical compound, drug | 20E (20-Hydroxyecdsone) | Cayman Chemical | Item No. 16145 |  |
|  |  |  |  |  |
|  |  |  | CAS No. 5289-74-7 |  |
|  |  |  |  |  |
| chemical compound, drug | Octopamine (OA) | Sigma-Aldrich | CAS No. 770-05-8 |  |
| chemical compound, drug | Ionomycin (Iono) | Cayman Chemical | Item No. 10004974  CAS No. 56092-81-0 |  |
|  |  |  |  |  |
|  |  |  |  |  |
|  |  |  |  |  |
| chemical compound, drug | L-012 | Wako Chemical | Cas No. 143556-24-5 |  |
|  |  |  |  |  |
|  |  |  |  |  |
| antibody | anti-Hnt | DSHB | Cat#1G9 | IF (1:75) |
|  | (Mouse monoclonal) |  | AB_528278 RRID:AB_528278 |  |
| antibody | anti-Cut | DSHB | Cat#2B10 | IF (1:15) |
|  | (Mouse monoclonal) |  | AB_528186 RRID:AB_528186 |  |
| antibody | anti-Br-C (Mouse monoclonal) | DSHB | Cat#25E9.D7 | IF (1:15) |
|  |  |  | AB_528104 RRID:AB_528104 |  |
| antibody | anti-EcR.A (Mouse monoclonal) | DSHB | Cat#15G1a | IF (1:30) |
|  |  |  | AB_10659792 |  |
|  |  |  | RRID:AB_10659792 |  |
| antibody | anti-EcR.B1 (Mouse monoclonal) | DSHB | Cat# AD4.4 | IF (1:30) |
|  |  |  | AB_2154902 RRID:AB_2154902 |  |
| antibody | anti-Ftz-f1 (Rabbit polyclonal) | gift from Dr. Hitoshi Ueda, Okayama University, Japan | RRID:AB_2569929 | IF (1:50000) |
| antibody | anti-Ttk69 (Rabbit polyclonal) | a gift from Dr. Wanzhong Ge, Zhejiang University, China |  | IF (1:100) |
| antibody | anti-GFP (Rabbit polyclonal) | Invitrogen | AB_221569 ; RRID:AB_221569 | IF (1:4000) |
| antibody | anti-GFP | Invitrogen | AB_221568; RRID:AB_221568 | IF (1:500) |
|  | (Mouse monoclonal) |  |  |  |
| antibody | anti-RFP | MBL international | AB_591279 RRID:AB_591279 | IF (1:2000) |
|  | (Rabbit polyclonal ) |  |  |  |
| antibody | anti-β-Gal | Abcam | Cat# ab9361 AB_307210 RRID:AB_307210 | IF (1:500) |
|  | (Chicken polyclonal) |  |  |  |
| antibody | anti-Sim (Guinea-pig polyclonal) | from Dr. Stephen Crews, University of North Carolina at Chapel Hill School of Medicine, Chapel Hill, USA |  | IF (1:1000) |
| antibody | Alexa Fluor 488 anti-Guinea pig (Goat polyclonal) | Invitrogen | AB_2534117 RRID:AB_2534117 | IF (1:1000) |
| antibody | Alexa Fluor 488 anti-Rabbit (Goat polyclonal) | Invitrogen | AB_143165 RRID:AB_143165 | IF (1:1000) |
| antibody | Alexa Fluor 488 anti-Mouse (Goat polyclonal) | Invitrogen | AB_2534069 RRID:AB_2534069 | IF (1:1000) |
| antibody | Alexa Flour 568 anti-chicken (Goat polyclonal) | Invitrogen | AB_2534098 RRID:AB_2534098 | IF (1:1000) |
| antibody | Alexa Flour 568 anti-guinea pig (Goat polyclonal) | Invitrogen | AB_2534119 RRID:AB_2534119 | IF (1:1000) |
| antibody | Alexa Flour 568 anti-Mouse (Goat polyclonal) | Invitrogen | AB_144696 RRID:AB_144696 | IF (1:1000) |
| antibody | Alexa Flour 568 anti-Rabbit (Goat polyclonal) | Invitrogen | AB_10563566 RRID:AB_10563566 | IF (1:1000) |
| commercial assay or kit | Click-iT™ Plus EdU Cell Proliferation Kit for Imaging, Alexa Fluor™ 555 dye | Invitrogen | Cat# C10638 |  |
| Chemical compound, drug | TRIzol | Life Technologies | Cat #15596018 |  |
| commercial assay or kit | Direct-zol™ RNA MicroPrep Kit | Zymo Research |  |  |
| commercial assay or kit | using Illumina TruSeq Stranded mRNA Sample Preparation kit | Illumina |  |  |
| Software, algorithm | Sickle | Sickle | OMICS_01077 RRID:SCR_006800 |  |
|  |  |  |  |  |
| Software, algorithm | HISAT2 | HISAT2 | RRID:SCR_015530 | (Kim et al., 2015) |
| Software, algorithm | HTSeq-count | HTSeq-count | RRID:SCR_011867 | (Anders et al., 2015) |
| Software, algorithm | DESeq2 |  | RRID:SCR_015687 | (Love et al., 2014). |
| Software, algorithm | Prism 7 | Graphpad | RRID:SCR_002798 |  |
| Recombinant DNA reagent | pAG/MNase | Addgene | ID# 123461 RRID:Addgene_123461 |  |
|  | (Plasmid ) |  |  |  |
| commercial assay or kit | NEBNext Ultra II DNA Library Prep Kit (NEB) |  |  | ( Liu et al., 2018) |
| antibody | anti-FLAG M2 (Mouse monoclonal) | Sigma-Aldrich | Cat# F1804 AB_262044 RRID:AB_262044 | C&R: (1:250) |
| antibody | IgG1 Negative contronl clone Ci4 (Mouse monoclonal) | Sigma-Aldrich | AB_97846 RRID:AB_97846 | C&R: (1:125) |
| Software, algorithm | CUT&RUNTools workflow | This paper |  | modified from (Zhu et al., 2019) |
